# Inhibitory neuron map of sevoflurane induced neurotoxicity model in young primates

**DOI:** 10.3389/fncel.2023.1252782

**Published:** 2023-10-31

**Authors:** Yixuan Niu, Yanyong Cheng, Zhengjie Miao, Jinnan Xu, Hong Jiang, Jia Yan

**Affiliations:** Department of Anesthesiology, Shanghai Ninth People’s Hospital, Shanghai Jiao Tong University School of Medicine, Shanghai, China

**Keywords:** anesthesia, sevoflurane, rhesus macaques, somatostatin, neurotoxicity

## Abstract

**Introduction:**

Sevoflurane, one of the most commonly used anesthetic agents in children, may induce neuronal dysfunction and cognitive impairment. Exposure to sevoflurane might induce an imbalance between neural excitation and inhibition which could be a mechanism behind anesthesia-induced cognitive and affective dysfunctions. However, the underlying mechanisms remain unclear.

**Methods:**

In this study, we used two rhesus macaques in the control group, and one rhesus macaques in the anesthesia group. We employed single-nucleus RNA sequencing (snRNA-seq) technology to explore alterations in distinct types of inhibitory neurons involved in the long-term cognitive impairment caused by sevoflurane in young macaques.

**Results:**

Following sevoflurane treatment, an upregulation was observed in the SST+ inhibitory neuron in the LHX6+ neighborhood in the hippocampus of rhesus macaques. This alteration might impact brain development by influencing interneuron migration and maturation. Additionally, we proposed a novel classification of inhibitory neurons, defined by CNR1 and LHX6 applicable to both humans and macaques.

**Discussion:**

Our study proposed a novel classification of inhibitory neurons defined by LHX6 and CNR1, relevant in macaques and humans. We also provide evidence that sevoflurane upregulated the SST+ inhibitory neuron in the LHX6+ neighborhood in the hippocampus of rhesus macaques, which may underlie the potential neurotoxic effects induced by general anesthetics. Our results also offer a more reliable approach for studying the structure and function of the human brain.

## 1. Introduction

Annually, millions of children globally undergo surgical procedures under anesthesia (Kearns et al., 2021). The brain, being the principal target of general anesthetics, is crucial in effectuating anesthesia (Niu et al., 2022). Consequently, anesthetic exposure in developing brains is a critical health concern, prompting significant public attention and controversy (Kearns et al., 2021). Clinical studies suggest that children exposed to anesthesia and surgery may encounter an increased susceptibility to cognitive impairment and structural brain alterations (Davidson and Sun, 2018). Particularly, children below 4 years who have experienced three or more instances of anesthesia/surgery, as opposed to a single instance, may be at a higher risk of deficits in processing speed and fine motor abilities (Warner et al., 2021). Moreover, there are reports indicating cognitive impairment and neurotoxicity in young animals, such as non-human primates (NHPs) and rodents, resulting from general anesthetic exposure (Yan et al., 2020; Zhang et al., 2022).

Sevoflurane, the most commonly used anesthetic in children, is believed to function primarily by activating inhibitory receptors in the central nervous system (CNS), including γ-aminobutyric acid (GABA) type A and glycine receptors (Chen et al., 2022). According to a recent study, exposing rhesus macaques that are 6 weeks old to sevoflurane three times, each for a four-hour duration, mild visual memory deficits may manifest at a later stage, around the age of one (Raper et al., 2017). These rhesus macaques may also exhibit signs of anxiety when they reach 2 years of age (Raper et al., 2018). Given that sevoflurane’s anesthetic effects are mediated through the activation of inhibitory neurons, pivotal in regulating anxiety behavior and memory, we focused our investigation on these neurons (Courtin et al., 2014; Scheggia et al., 2020). Therefore, understanding the underlying mechanisms by which these neurons contribute to cognitive dysfunction due to sevoflurane anesthesia is crucial.

Within the human cerebral cortex, the inhibitory neurons are predominantly classified into two major branches, NR2F2 and LHX6, which are similar to those found in the cortex of mice (La Manno et al., 2016; Zeisel et al., 2018; Gouwens et al., 2020; Zhong et al., 2020). The LHX6 branch includes PVALB and SST subclasses, while the NR2F2 branch encompasses LAMP5, PAX6, and VIP subclasses (Liu et al., 2017). We employed snRNA-seq technology to examine the alterations in different types of inhibitory neurons associated with long-term cognitive impairment induced by sevoflurane in young macaques. A key finding of this study is the bifurcation of inhibitory neurons into two groups, differentiated by their expression of LHX6 and CNR1. Our results indicate that sevoflurane particularly impacts the SST subtype of inhibitory neurons within the LHX6 group. These findings could offer valuable insights into the role of inhibitory neurons in neurodevelopmental toxicity induced by general anesthetics.

## 2. Materials and methods

### 2.1. Anesthesia of rhesus macaque

The use of rhesus macaques in our study was approved by the Institutional Animal Care and Use Committee (protocol number, XC17001), and stringent measures were taken to minimize the number of animals. The rhesus macaques received sevoflurane anesthesia as was described in our previous studies (Zhang et al., 2019). The control group included two rhesus macaques and the anesthesia group one rhesus macaque. Briefly, in the anesthesia group, the rhesus macaques received 6–8% anesthetic sevoflurane with 100% oxygen for the induction (2–4 min) of general anesthesia. Subsequently, they received 2.5–3% sevoflurane and 100% oxygen for a duration of 4 h each day on postnatal day (P)7, P21, and P35. The respiratory amplitude and frequency were found to be normal. We monitored the blood levels of electrolytes, including sodium, potassium, and chloride, to ensure they remained within the normal range. The monitored heart rate and saturation of pulse oxygen (SpO2) were consistent with normal physiological parameters. No hypoxia or carbon dioxide accumulation was observed. In addition, the vital parameters of exposed macaques were also found to be in the normal range during previous studies under the same conditions, excluding the possibility of physiological disturbances affecting the experimental outcomes. Animal temperature was maintained by placing them in a warm box (37°C). After a 30-min recovery period from anesthesia, the rhesus macaques were returned to their mothers in the cage. The rhesus macaques in the control group received three maternal separations of the same duration (4 h). The rhesus macaques were sacrificed via decapitation under brief sevoflurane anesthesia (3% sevoflurane for 5 min) at the end of the third sevoflurane anesthesia on P35. Immediately afterward, the hippocampus of the rhesus macaques was harvested. For the PCR assays, three female rhesus macaques (namely, C1, C2, and C3) in the control group and one male and two female rhesus macaques in the anesthesia group (namely, S1, S2, and S3, respectively) were used.

### 2.2. Data download

scRNA-seq data analysis was performed by NovelBio Co., Ltd. with NovelBrain Cloud Analysis Platform.^1^ We downloaded the expression matrix (GSE131258) and used the data of three samples (GSM3360835, GSM3770749, GSM3770750) for subsequent analysis. Cells contained over 200 expressed genes and mitochondria UMI rate below 20% passed the cell quality filtering and mitochondria genes were removed in the expression table.

### 2.3. Nucleus isolation

Single-nucleus RNA-seq experiment was performed in the laboratory of NovelBio Bio-Pharm Technology Co., Ltd. We collected nuclei cells from the hippocampus of three postnatal 35 days old (P35) rhesus macaques and performed single nucleus RNA sequencing (snRNA-seq) repetitively for eight times with samples combined from the three macaques. The tissues samples were surgically removed and snap frozen in liquid nitrogen for intact nucleus isolation. The nucleus was isolated and purified as previously described with some modifications (Krishnaswami et al., 2016). Briefly, the frozen tissue was homogenized in NLB buffer which contain 250 mM Sucrose, 10 mM Tris–HCl, 3 mM MgAc2, 0.1% Triton X-100 (SigmaAldrich, USA), 0.1 mM EDTA, 0.2 U/μL RNase Inhibitor (Takara, Japan). Various concentration of sucrose was used to purify the nucleus. The concentration of nucleus was adjusted to about 1,000 nuclei/μL for snRNA-Seq.

### 2.4. Single-nucleus RNA-seq experiment

The snRNA-Seq libraries were generated using the 10X Genomics Chromium Controller Instrument and Chromium Single Cell 3’ V3 Reagent Kits (10X Genomics, Pleasanton, USA). Briefly, cells nuclei were concentrated to approximately 1,000 nuclei/μL than loaded into each channel to generate single-cell Gel Bead-In-Emulsions (GEMs). After the RT step, GEMs were broken and barcoded-cDNA was purified and amplified. The amplified barcoded cDNA was fragmented, A-tailed, ligated with adaptors and index PCR amplified. The final libraries were quantified using the Qubit High Sensitivity DNA assay (Thermo Fisher Scientific, USA) and the size distribution of the libraries were determined using a High Sensitivity DNA chip on a Bioanalyzer 2,200 (Agilent, USA). All libraries were sequenced by Novaseq6000 (Illumina, USA) on a 150 bp paired-end run.

### 2.5. Single-nucleus RNA statistical analysis

snRNA-seq data analysis was performed by NovelBio Bio-Pharm Technology Co., Ltd. with NovelBrain Cloud Analysis Platform (see text footnote 1). We applied fastp with default parameter filtering the adaptor sequence and removed the low quality reads to achieve the clean data. Then the feature-barcode matrices were obtained by aligning reads to the common marmoset genome (ASM275486v1_Ensembl104) using CellRanger v6.1.1. We applied the down sample analysis among samples sequenced according to the mapped barcoded reads per cell of each sample and finally achieved the aggregated matrix. Cells contained over 200 expressed genes passed the cell quality filtering were removed in the expression table.

Seurat package (version: 4.0.3)^2^ was used for cell normalization and regression based on the expression table according to the UMI counts of each sample to obtain the scaled data. PCA was constructed based on the scaled data with top 2000 high variable genes and top 10 principals were used for tSNE construction and UMAP construction. Utilizing graph-based cluster method, we acquired the unsupervised cell cluster result based the PCA top 10 principal and we calculated the marker genes by FindAllMarkers function with wilcox rank sum test algorithm under following criteria:(1) log2FC > 0.25; (2) *p*value<0.05; (3) min.pct > 0.1. In order to identify the cell type detailed, the clusters of same cell type were selected for re-tSNE analysis, graph-based clustering and marker analysis.

A total of 52,690 cells were obtained for our classification analysis. Of these, 9,715 cells were used in an astrocyte-type analysis; 1,185 cells in an endothelial and pericyte-type analysis; 20,048 cells in an excitatory neuron-type analysis; 7,604 cells in an inhibitory neuron-type analysis; 3,134 cells in a microglia-type analysis; and 11,004 cells in an OPC&OL-type analysis. After sevoflurane anesthesia, a reduction was observed in the numbers of microglial cells, OPC&OL cells, and astrocyte cells. Additionally, there was an increase in the numbers of endothelial and pericyte cells and excitatory neurons.

### 2.6. GO analysis

Gene ontology (GO) analysis was performed to facilitate elucidating the biological implications of marker genes and differentially expressed genes. We downloaded the GO annotations from NCBI^3^, UniProt^4^ and the Gene Ontology^5^. Fisher’s exact test was applied to identify the significant GO categories and FDR was used to correct the *p*-values.

### 2.7. Pathway analysis

Pathway analysis was used to find out the significant pathway of the marker genes and differentially expressed genes according to KEGG database. We turn to the Fisher’s exact test to select the significant pathway, and the threshold of significance was defined by *p*-value and FDR.

### 2.8. Quantitative real-time PCR

The total RNA of the hippocampus of the rhesus macaques after receiving sevoflurane anesthesia was harvested with TRIzol (Invitrogen, USA) according to the manufacturer’s instructions. Total RNA was reverse transcribed into cDNA using the Prime-Script RT Reagent Kit (Takara, Japan). The standard SYBR-Green method was used to detect the relative expression of SST, KCNIP4, RGS6, SPARCL1, and HBSL1 with Lightcycler 480 (Roche, USA). GAPDH is used for reference gene for normalize q-PCR. The relative expression of target genes is presented as 2 − ΔΔCt.

Primers for the qPCR detection are listed as follows:

SST

PF 5’-CCAACCAGACGGAGAATGATGCC-3’

PR 5’-GGTGCCATAGCTGGGTTTGAGTTAG-3’

KCNIP4

PF 5’-GCGTGGAAGATGAACTGGAGATGG-3’

PR 5’-AACAACACCACTGGGGCATTCG-3’

RGS6

PF 5’-AGACGCCCAGGAGCACATCTAC-3’

PR 5’-CGCCAGCGACTTTCCCTTCTTC-3’

SPARCL1

PF 5’-TGATGATGATGGCGGTGATGATGG-3’

PR 5’-TGAGGTGATAGGCAATGGATTGAGC-3’

HBSL1

PF 5’-ACGGTTCAAGCATCAGAAGAGCAG-3’

PR 5’-GACCAATGACCACCAAGTTGAGGAG-3’

### 2.9. Statistics

All values were presented as mean ± standard errors (SEM). All the data were analyzed by one-way ANOVA analysis of variance followed by the Tukey’s test. The statistical analysis was performed using GraphPad Prism 9.0 (GraphPad, USA) software. Statistical significance was accepted as *p* < 0.05.

## 3. Results

### 3.1. A novel classification of inhibitory neurons was proposed which was defined by CNR1 and LHX6

**Figure 1:**
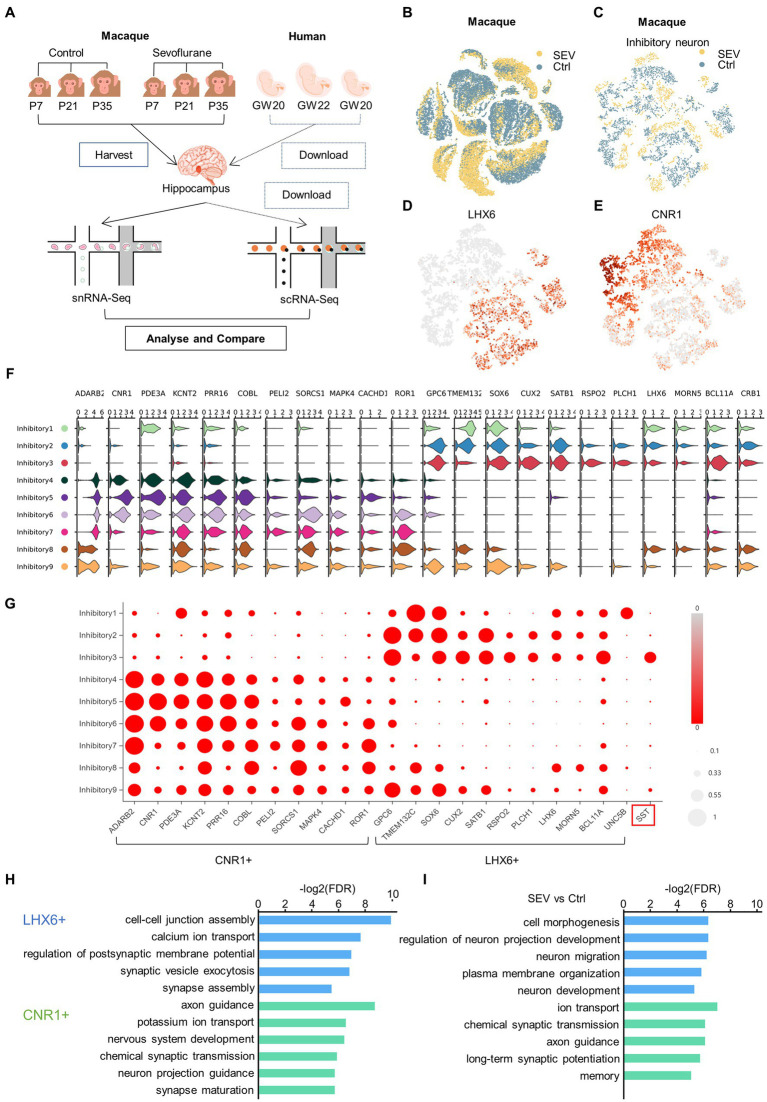
A novel classification of inhibitory neurons was proposed which was defined by CNR1 and LHX6. **(A)** Experimental design and workflow. There were two rhesus macaques in the control group (Ctrl), and one rhesus macaque in the anesthesia group (SEV), which received sevoflurane anesthesia at P7, P21 and P35. The hippocampus of the rhesus macaques were harvested at the end of the third sevoflurane anesthesia for single-nucleus RNA-seq. The three human fetal hippocampus scRNA-seq data GSE119212 was downloaded from the GEO database. Zhong et al. (2020) analysed cells from the entire left hippocampus at gestational weeks (GW) by droplet-based single-cell RNA sequencing. **(B)** t-Distributed stochastic neighbor embedding (t-SNE) of the two groups in the developing macaque hippocampus, colored by group (yellow, SEV; blue, Ctrl). **(C)** t-SNE visualization of two groups of inhibitory neuron in the developing macaque hippocampus, colored in group (yellow, SEV; blue, Ctrl). **(D, E)** The neighborhood (i.e., the upstream level) of clusters, were defined by LHX6 **(D)** and CNR1 **(E)**. t-SNE shows different neighborhood of clusters between control and sevoflurane. **(F, G)** Inhibitory neurons were clearly categorized into the CNR1+ neighborhood and the LHX6+ neighborhood according to DEGs, visualized in violin plot and bubble chart. **(H)** GO enrichment of LHX6+ and CNR1+ neighborhood of cluster compared to each other, demonstrating original different biological functions between LHX6+ and CNR1+ neighborhood of cluster. **(I)** GO enrichment of DEGs in LHX6+ and CNR1+ neighborhood of cluster compared to each other, illustrating the different biological functions between control condition and sevoflurane.

To define cell types in primates’ hippocampus during development, we analyzed cells from the macaque hippocampus using snRNA-seq visualized in t-SNE, and also utilized human fetal hippocampus scRNA-seq data sourced from the GEO database (Figures 1A–C). We collected the nuclei cells from the hippocampus of macaques and performed snRNA-seq eight times, using samples combined from three individual macaques. Based on the snRNA-seq results and existing classifications, we proposed a novel classification of inhibitory neurons.

Neurons can be divided into two major classes: cortical plate-derived excitatory neurons and ganglionic eminence-derived inhibitory neurons (Hodge et al., 2019). The inhibitory neurons can be further subclassified as deriving from either the medial or caudal ganglionic eminence (MGE or CGE), based on LHX6 and NR2F2 expression (Zhong et al., 2020). We defined the neighborhood as the upstream level of the cluster. Within the macaque hippocampus, inhibitory neurons can be separated into two neighborhoods, defined by CNR1 and LHX6 expression (Figures 1D,E). Inhibitory neurons were clearly categorized into two neighborhoods according to differentially expressed genes (DEGs) CNR1, ADARB2, PDE3A, KCNT2, PRR16, COBL, PELI2, SORCS1, MAPK4, CACHD1, and LHX6, GPC6, TMEM132C, SOX6, CUX2, SATB1, RSPO2, PLCH1, MORN5, BCL11A, CRB1 (Figures 1F,G). Gene ontology (GO) analysis of the DEGs highlighted different biological functions between the two neighborhoods, both pre- and post-sevoflurane exposure. Post-exposure, the biological functions of the LHX6+ neighborhood were primarily associated with cell morphogenesis, regulation of neuron projection development, neuron migration, plasma membrane organization, and neuron development. Concurrently, the biological functions of the CNR1+ neighborhood were enriched in areas such as ion transport, chemical synaptic transmission, axon guidance, long-term synaptic potentiation, and memory (Figures 1H,I).

### 3.2. Sevoflurane upregulated the SST+ inhibitory neuron in the LHX6+ neighborhood

Sevoflurane, one of the most frequently used anesthetic agents in children, could potentially induce neurodevelopmental and cognitive dysfunctions in rodents and primates (Zhang et al., 2022). The hippocampus harbors both excitatory and inhibitory neurons, with sevoflurane exerting its anesthetic effect by inhibiting excitatory neurons and activating inhibitory ones (Niu et al., 2022). The delicate balance between excitation and inhibition is crucial for normal brain function (Xu et al., 2019). Any disturbance to this balance, such as that potentially caused by sevoflurane exposure, might contribute to the cognitive and affective dysfunction seen with anesthesia (Zhao et al., 2020).

**Figure 2:**
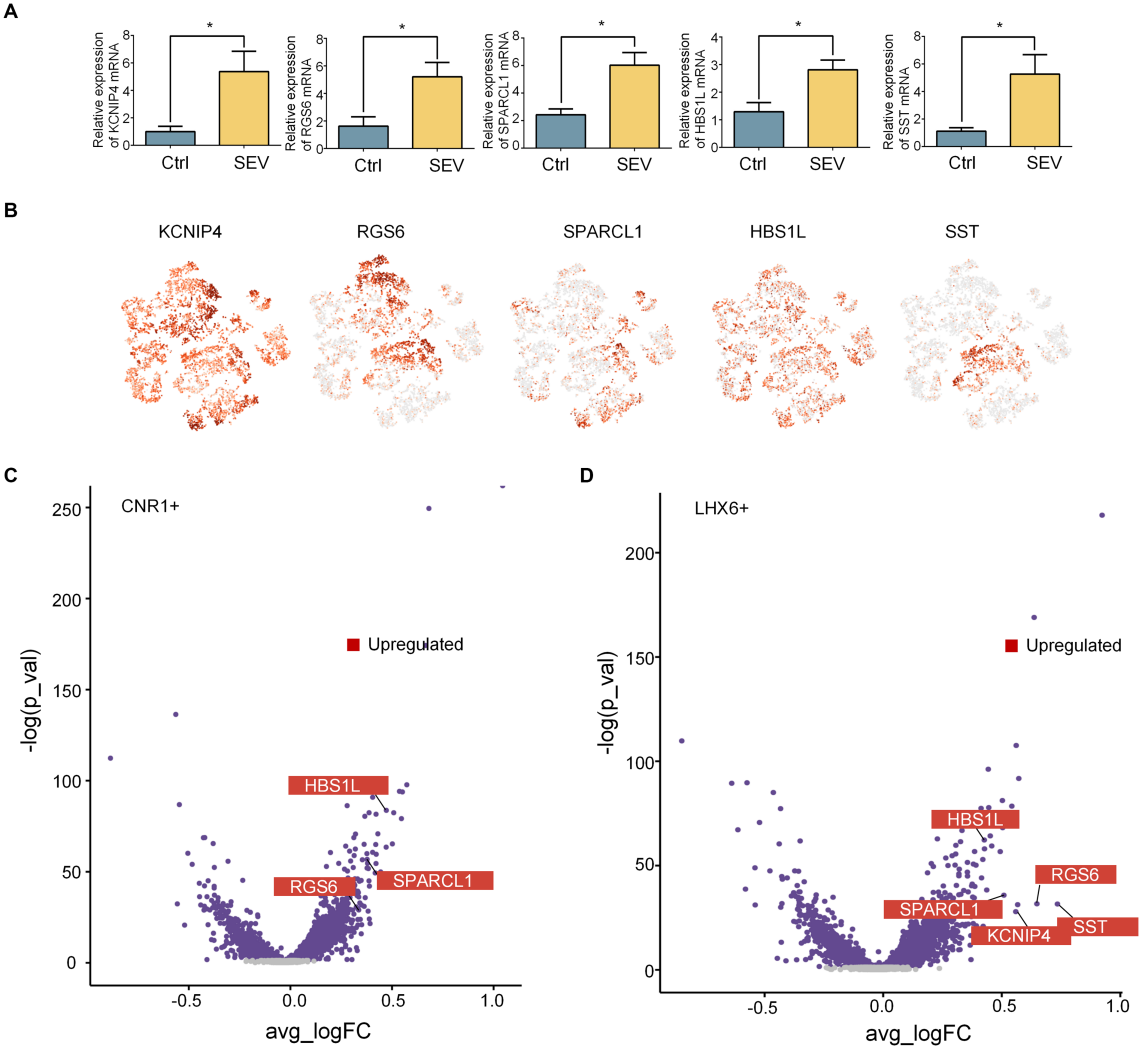
Sevoflurane upregulated the SST+ inhibitory neuron in the LHX6+ neighborhood. **(A)** qPCR revealed the upregulation of SST (*n* = 3, *p* = 0.0443), KCNIP4 (*n* = 3, *p* = 0.0470), RGS6 (*n* = 3, *p* = 0.0447), SPARCL1 (*n* = 3, *p* = 0.0236), and HBS1L (*n* = 3, *p* = 0.0349) after sevoflurane treatment (SEV vs. Ctrl) in the hippocampus of macaques. Data are represented as mean ± SEM, *p < 0.05. **(B)** Expression levels of SST, KCNIP4, RGS6, SPARCL1, and HBS1L in the inhibitory neuron in the macaque hippocampus, using t-SNE visualization. **(C, D)** Volcano plot showing the changes in the DEGs in LHX6+ neighborhood and CNR1+ neighborhood of inhibitory neurons after sevoflurane exposures. Sevoflurane caused diverse changing pattern in different neighborhoods.

Inhibitory neurons can be subclassified as deriving from either the MGE or CGE, based on the expression of LHX6 and NR2F2, and MGE generates SST+ hippocampal interneurons (Zhong et al., 2020). Our research confirmed that sevoflurane treatment leads to upregulation of KCNIP4, RGS6, SPARCL1, HBS1L, and SST in the rhesus macaque’s hippocampus, as detected by qPCR (Figure 2A). While the t-SNE indicated that KCNIP4, RGS6, SPARCL1, and HBS1L exhibited scattered distributions, SST showed a more concentrated distribution. Comparatively, the t-SNE plot in Figure 1 highlighted that SST was predominantly concentrated within the LHX6+ neighborhood (Figure 2B). As compared to the control group (Ctrl), three genes were upregulated in the CNR1+ neighborhood and five in the LHX6+ neighborhood (Figures 2C,D). These upregulated genes are consistent with our PCR findings. Following sevoflurane exposure, the SST gene displayed an increased specificity in the LHX6+ neighborhood and did not significantly change in the CNR1+ neighborhood, suggesting that anesthesia neurotoxicity of sevoflurane might be mediated by the activation of the SST+ inhibitory neurons in the LHX6 neighborhood, other than CNR1.

Somatostatin, a cyclic neuropeptide, modulates a variety of physiological functions, including sleep, motor activity, sensory functions, emotions, as well as learning and memory. It is also involved in diverse pathological conditions, such as pain, inflammation, neurodegeneration, anxiety, and depression (Song et al., 2021). LHX6, a protein-coding gene, acts as a transcription factor required for expressing a subset of genes involved in interneuron migration and development. It is associated with the specification of cortical interneuron subtypes and the migration of GABAergic interneuron precursors from the subpallium to the cerebral cortex (Yuan et al., 2020). Our findings suggest that sevoflurane may boost SST+ inhibitory neurons in the LHX6+ neighborhood, thereby affecting brain development by impacting interneuron migration and development and disrupting the excitation-inhibition balance.

### 3.3. The role of SST in synapse and neuron development

The SST gene is widely expressed during embryonic development and encodes somatostatin, which is an important inhibitory neurotransmitter (Song et al., 2021). In the developing brain, SST regulates neuronal development and migration while playing a critical role in synaptic formation and maturation (Su et al., 2020).

**Figure 3:**
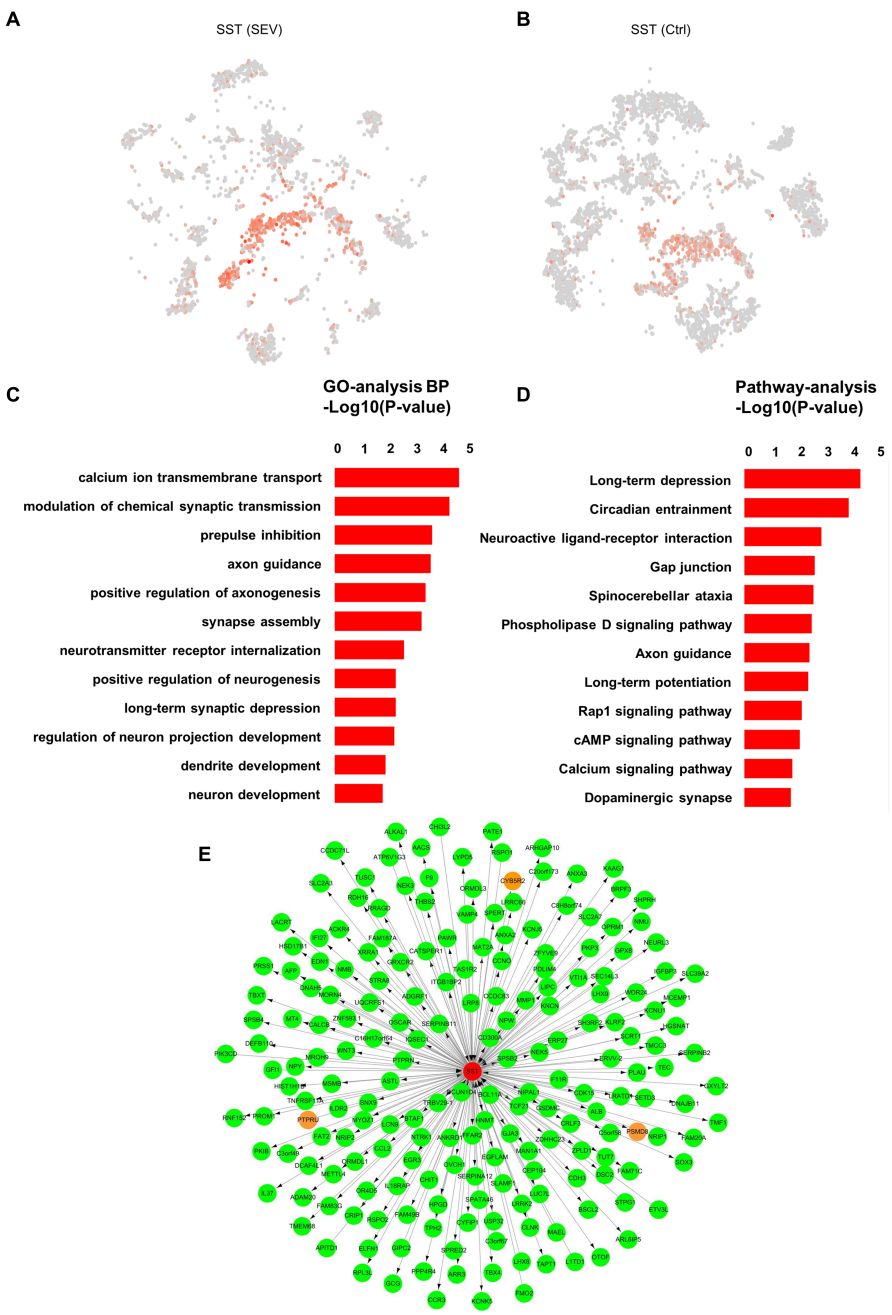
The role of SST in synapse and neuron development. **(A, B)** Visualization of the expression level of SST in the inhibitory neuron in the macaque hippocampus pre- and post-sevoflurane treatment, using t-SNE visualization. **(C)** Gene Ontology (GO) enrichment analysis of the genes expressed in the SST+ cluster between two groups (Ctrl vs. SEV). **(D)** KEGG pathway analysis of the genes expressed in the SST+ cluster between two groups (Ctrl vs. SEV). **(E)** Weighted gene co-expression network analysis (WGCNA) was performed in order to build the co-expression network.

We have confirmed the upregulation of SST gene expression by snRNA-seq and PCR (Figures 3A,B). However, what could be the implications of the upregulated SST expression? This is the question we aimed to address. Moreover, GO analysis suggested a strong association of the SST gene with functions such as calcium ion transmembrane transport, synaptic transmission, neurogenesis, and neuron development (Figures 3C,D). We performed weighted gene co-expression network analysis (WGCNA) in order to build the co-expression network of SST (Figure 3E). Neuronal communication relies on synapses, with calcium ions serving as essential signaling molecules for neuronal synaptic activity (Platholi and Hemmings, 2022). Previous studies reported that general anesthetics could affect synaptic plasticity by influencing calcium ion transmembrane transport (Platholi and Hemmings, 2022). The GO results suggest that sevoflurane might disrupt neuronal synapse formation and development by impacting calcium ion transmembrane transport. Such interference can impede communication and information exchange between neurons, thereby affecting the normal establishment and functionality of neural networks, ultimately resulting in cognitive, developmental, and social impairments.

Moreover, neurogenesis, particularly dendritic spine morphogenesis, is a vital step in brain circuit development within the central nervous system (Liao et al., 2021). During the peak period of brain development, the abnormal morphological plasticity of dendritic spines can trigger disruptions in synapse formation, leading to long-term neurodevelopmental dysfunction (Bian et al., 2015). Our earlier study suggested that exposure to 2% sevoflurane for 3 h induces abnormal dendritic spine morphological proportions (but not density) and could result in abnormal neurogenesis, potentially influencing developmental brain injury (Zhong et al., 2022). Therefore, a comprehensive understanding of the SST gene’s role is essential to provide a stronger foundation for fundamental research on the safety of infant brain development during anesthesia.

### 3.4. Classification of neurons defined by LHX6 and CNR1 in macaques is applicable in humans

**Figure 4:**
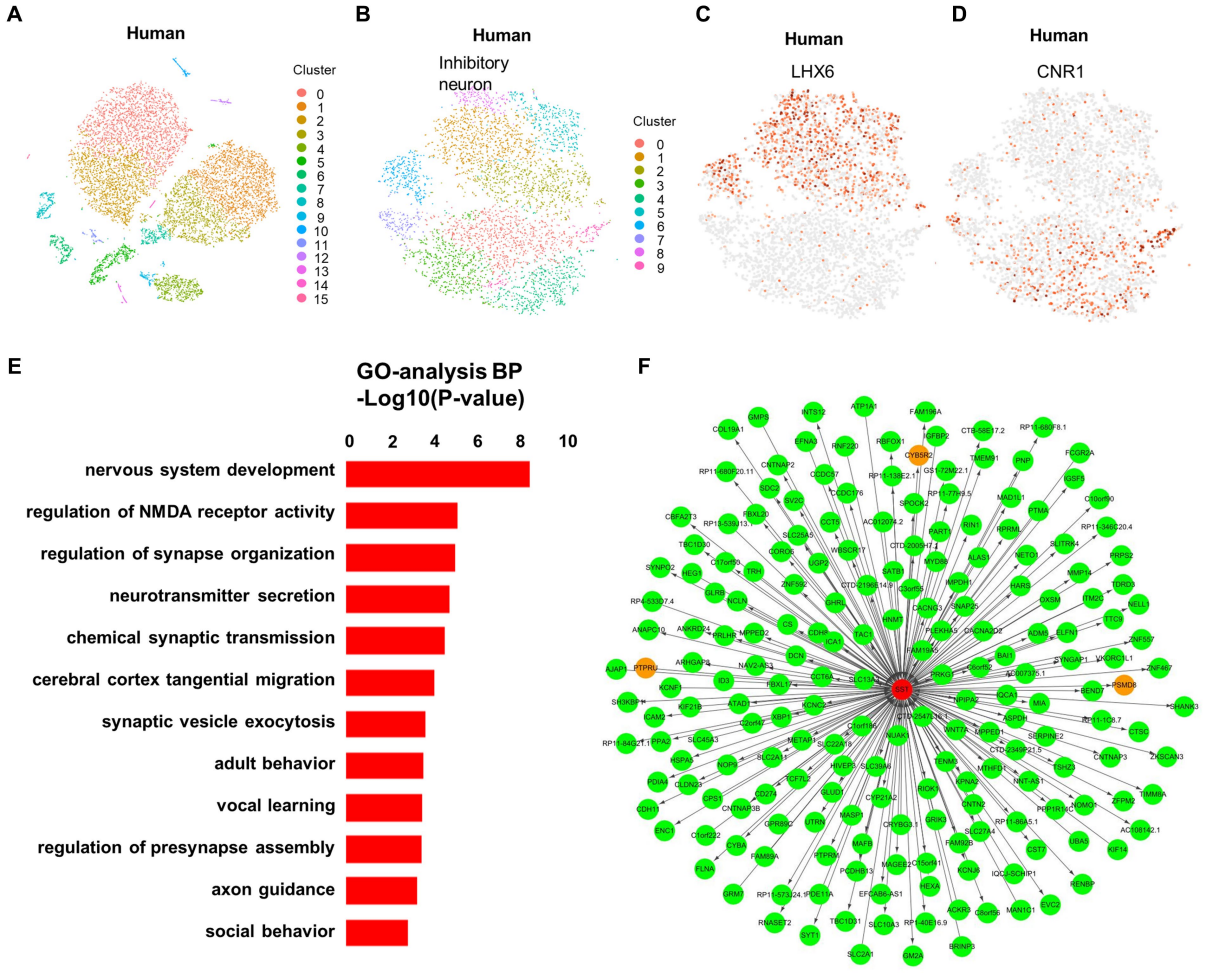
Classification of neurons defined by LHX6 and CNR1 in macaques is applicable in humans. **(A)** Visualization of 16 major clusters in the developing human fetus hippocampus using t-SNE visualization, colored in clusters. **(B)** Ten subclusters of the inhibitory neuron in the developing human fetus hippocampus using t-SNE visualization, colored in clusters. **(C, D)** The neighborhood (i.e., the upstream level) of clusters in the developing human fetus, were defined by LHX6 **(C)** and CNR1 **(D)**. **(E)** GO enrichment analysis of the genes expressed in the SST+ cluster of the developing human fetus. **(F)** Weighted gene co-expression network analysis (WGCNA) was performed in order to build the co-expression network.

In our study, we proposed a unique neuronal classification, based on the expression of LHX6 and CNR1, applicable not only in macaques but also in humans. This classification is visualized using t-SNE (Figures 4A–D). The transcription factor LHX6 plays a critical role in the development of GABAergic interneurons, while CNR1 serves as an endocannabinoid receptor that modulates neurotransmitter release (Asgarian et al., 2022; Claypool et al., 2022). The expression of these two genes proves to be critical in the classification of neuronal types and subtypes (Zhong et al., 2020). In macaques, we can use a clustering analysis of the expression levels of these two genes to classify neurons into different categories. This enables us to more accurately determine the neuronal types and subtypes in different regions, and to investigate the functional differences and interactions among different neuronal types in aspects such as human cognition and behavior. Such knowledge holds great importance for cognitive neuroscience, neurological disease research, and related fields. Given the challenges in obtaining samples from developing human brains, this gene-expression-based neuronal classification proves advantageous as it is also applicable in humans, thereby providing a reliable foundation for studying the human brain’s structure and function.

In addition, GO analysis showed that the SST gene is associated with nervous system and synapse development in infant brains (Figure 4E). This result aligns closely with the function of the SST gene in macaque brains, suggesting potential similar alterations in the human brain. Furthermore, identifying the upstream and downstream genes of the SST gene via WGCNA can reveal the signaling pathways and biological processes regulated by the SST gene, thereby enhancing our understanding of SST gene’s role in neuronal development, function, and disease (Figure 4F). This is of great importance for investigating the pathogenesis of neurological disorders and developing new therapeutic strategies.

## 4. Discussion

In this study, we have introduced a novel classification of inhibitory neurons, defined by the expression of LHX6 and CNR1, applicable to both macaques and humans. Furthermore, we found that sevoflurane might upregulate SST expression within the LHX6+ neighborhood in the hippocampus of rhesus macaques. This could potentially influence brain development by modulating synapse and neuron development.

Numerous studies suggest that several commonly used general anesthetics could lead to cognitive dysfunctions in adulthood (Alvarado et al., 2017; Raper et al., 2018; Zhang et al., 2022; Zhong et al., 2022). Nonetheless, the practical challenges of acquiring pediatric human brain samples necessitate the identification of suitable animal models to investigate the safety of anesthetics on brain development. Given that NHPs share 98% genetic similarity with humans, compared to rodents, they can often more accurately predict the human body’s responses to pathological conditions (Wang et al., 2017). Therefore, we selected NHPs as our research model. Additionally, the considerable changes that various cell types undergo during early developmental stages underscore the importance of identifying a molecular marker that can serve as a temporal indicator throughout development. Our findings suggest that LHX6 may serve as such a marker. However, when validated using human data, this classification seemed less effective than the previously reported classification in human fetuses based on LHX6 and NR2F2 (Zhong et al., 2020). This outcome suggests that while LHX6 is consistent across different developmental stages in humans and macaques, CNR1 and NR2F2 may vary between the two species due to species-specific or developmental stage differences. Therefore, we proposed a novel classification that distinguishes itself by its applicability in both humans and macaques, providing a robust supplement for future investigations.

This novel classification facilitates a more accurate identification and categorize inhibitory neurons, thereby facilitating a more comprehensive understanding of their characteristics and functions. During the early neuronal development stages, a balance between excitatory and inhibitory neurons within the central nervous system is essential for maintaining proper brain function (Zhao et al., 2020). However, sevoflurane might enhance the number of SST+ inhibitory neurons, potentially leading to an imbalance between excitatory and inhibitory neurons (Zhao et al., 2020). Numerous studies have linked inhibitory neuron dysfunction to various psychiatric disorders, including social and processing dysfunction, anxiety, depression, schizophrenia, and autism (Yizhar et al., 2011; Marín, 2012). In this study, we noted that sevoflurane particularly impacts the SST+ inhibitory neurons in the LHX6+ neighborhood. SST+ inhibitory neurons, a specific subset of inhibitory neurons, primarily release the inhibitory neurotransmitter SST, playing an important role in regulating neural functions such as learning and memory (Hamm and Yuste, 2016). Furthermore, these neurons are critical to brain development, particularly in controlling synapse formation and neuron migration (Su et al., 2020). Our findings suggest that SST upregulation might disrupt the balance of excitatory and inhibitory neurons in the central nervous system, potentially contributing to the neurotoxicity induced by general anesthetics.

A previous study has revealed that, while multiple exposures to sevoflurane during the neonatal period resulted in long-term cognitive impairments in both male and female mice, there were significant sex-specific differences in their snRNA-seq (Song et al., 2023). In male mice, sevoflurane reduced the number of ligand-receptor pairs significantly enriched in the tri-synaptic loop of the hippocampus, reduced neuronal diversity in the CA1 region of the hippocampus, impaired the differentiation capacity of astrocytes in the hippocampus, and inhibited neuroregeneration in granule cells within the DG area. However, the impact on these functions in female mice was minimal (Song et al., 2023). Multiple exposures to sevoflurane during the neonatal period specifically increased the differentiation capacity of oligodendrocytes in the hippocampus in female mice, accompanied by increased expression of the Mbp and Mag genes (Song et al., 2023). In this study, we did not assess gender differences due to the limited number of macaques. Further research is warranted to explore sex differences in the effects of general anesthesia in juvenile non-human primates.

In our study, we collected nuclei cells from the hippocampus of macaques and performed snRNA-seq. However, the prefrontal cortex, which governs super-neurological functions, plays a crucial role in sevoflurane-induced neurodevelopmental toxicity. The prefrontal cortex and the hippocampus share some similarities. For instance, both are vital neural structures in the brain and are crucial for normal emotion, social interaction, perception, and attention (Zhu et al., 2023). However, the prefrontal cortex and the hippocampus also exhibit certain distinctions. The medial prefrontal cortex subregions have long been thought to play a critical role in emotional and social processing, whereas the hippocampus is primarily involved in the formation of short-term and long-term memories (Zhu et al., 2023). In future studies, we intend to further investigate the specific roles and interactions of the prefrontal cortex and the hippocampus in neurodevelopmental toxicity induced by general anesthetics.

We observed an increase in SST inhibitory neurons in our study, which may lead to an excitatory-inhibitory imbalance. Previous study has indicated that exposing 6-week-old rhesus macaques to sevoflurane 3 times, each for a 4 h duration, may result in mild visual memory deficits emerging at a later stage, typically around 1 year of age (Raper et al., 2017). Additionally, these rhesus macaques may display signs of anxiety when they reach 2 years of age (Raper et al., 2018). Our study’s findings are consistent with behavioral changes observed in macaques.

Previous study indicated that both propofol and sevoflurane had substantial and transient effects on the transcriptome profiles of microglia, excitatory neurons, interneurons, astrocytes, and oligodendrocyte progenitor cells, showed that clinically relevant doses of both propofol and sevoflurane promoted “microgliosis,” but only sevoflurane induced significant alterations in microglial gene expression patterns (Chang et al., 2023). Our study used snRNA-seq in rhesus macaques and compared the data with publicly available human embryonic data from databases, aims to help explain the potential neurotoxicity induced by general anesthetics and provide a blueprint for future mechanistic studies of anesthetics, which shares some similarities with the mentioned publication. Previous publication also conducted KEGG pathway analysis of differently expressed genes in inhibitory neurons after sevoflurane exposure. The results indicated that differently expressed genes were enriched in pathways related to WNT signaling pathway, viral carcinogenesis, and platelet activation, after 6 h of sevoflurane anesthesia (Chang et al., 2023). Notably, the WNT signaling pathway plays a crucial role in the nervous system, exerting significant influence on neuronal development and synaptic formation (Lv et al., 2023). Consistent with the previously described effects of sevoflurane on inhibitory neurons, which were associated with neuronal development and synaptic formation, the results in our study suggested a strong association of the SST inhibitory neurons with functions such as synaptic transmission, neurogenesis, neuron development, and calcium ion transmembrane transport.

However, some distinctions still exist between the current and previous findings. Previous publication used the human fetal prefrontal cortex (PFC) mixed cell cultures, while we used the rhesus macaque hippocampus. In the developing human brain, the inhibitory neurons can be subclassified as being derived from the medial or caudal ganglionic eminence (MGE or CGE) on the base of LHX6 and NR2F2 expression (Zhong et al., 2020). This is not entirely congruent with the classification in our study. Based on the results of our study and the existing classification systems, we proposed a novel classification approach. However, upon validation using human data, we observed that the performance of this classification method was inferior to the previously reported LHX6 and NR2F2 classification approaches as previously reported (Zhong et al., 2020). This suggests that LHX6 exhibits commonality across different developmental stages in both humans and primates, but the CNR1 and NR2F2 classification methods may exhibit inter-species or temporal variability between humans and primates. Given the substantial variation among cell categories during early developmental stages, identifying a universal marker along the developmental timeline becomes crucial. LHX6 may potentially serve as such a marker.

This study has several limitations. First, the rhesus macaque experiment was conducted with a limited sample size. However, obtaining samples from rhesus macaques is very challenging, making them highly valuable. Despite this limitation, our research findings contribute valuable insights for the study of human brain development and hold significant academic value. Increasing the sample size in future studies might be beneficial. Second, the age stages of humans and macaques within this study do not align perfectly. Despite this, all of them were in a developmental state, and we identified a consistent classification between the two, underscoring the significant academic value of our findings. Lastly, it is important to note that our functional investigation is solely dependent on the GO analysis and lacks behavioral validation. Future studies will specifically focus on SST+ neurons and incorporate behavioral validation in NHPs.

In conclusion, we proposed a novel classification of inhibitory neurons, defined by LHX6 and CNR1, applicable to both macaques and humans. Furthermore, this study found that sevoflurane may upregulate the SST+ inhibitory neuron within the LHX6+ neighborhood in the hippocampus of rhesus macaque. This observation could potentially explain the potential neurotoxicity induced by general anesthetics.

## Data availability statement

The datasets presented in this study can be found in online repositories. The names of the repository/repositories and accession number(s) can be found at: GSE153598 (GEO).

## Ethics statement

The studies involving humans were approved by the human data was downloaded from the GEO database, GSE119212. The studies were conducted in accordance with the local legislation and institutional requirements. Written informed consent for participation in this study was provided by the participants’ legal guardians/next of kin. The animal study was approved by the use of rhesus macaques in our study was approved by the Institutional Animal Care and Use Committee (protocol number, XC17001). The study was conducted in accordance with the local legislation and institutional requirements.

## Author contributions

JY and HJ: conceptualization. YC and YN: methodology and writing—original draft preparation. YN, YC, ZM, JX, HJ, and JY: writing—review and editing. All authors contributed to the article and approved the submitted version.

## Footnotes

^1^www.novelbrain.com

^2^https://satijalab.org/seurat/

^3^http://www.ncbi.nlm.nih.gov/

^4^http://www.uniprot.org/

^5^http://www.geneontology.org/
