# Supplementary material for: Inhibitory neuron map of sevoflurane induced neurotoxicity model in young primates
Source: Front Cell Neurosci. 2023 Oct 31;17:1252782. doi: 10.3389/fncel.2023.1252782 (PMC10643782; doi:10.3389/fncel.2023.1252782)
Supplement: Supplementary file 1 [file Data_Sheet_1.docx]

Supplementary Material

Novel Classification of Inhibitory Neurons Defined by CNR1 and LHX6 is Proposed Which is Applicable in Both Humans and Macaques

Yixuan Niu1, Yanyong Cheng1, Jia Yan1*

*** Correspondence:** Jia Yan: mzkyanj@163.com

# Supplementary Figures

**Figure S1.**

**
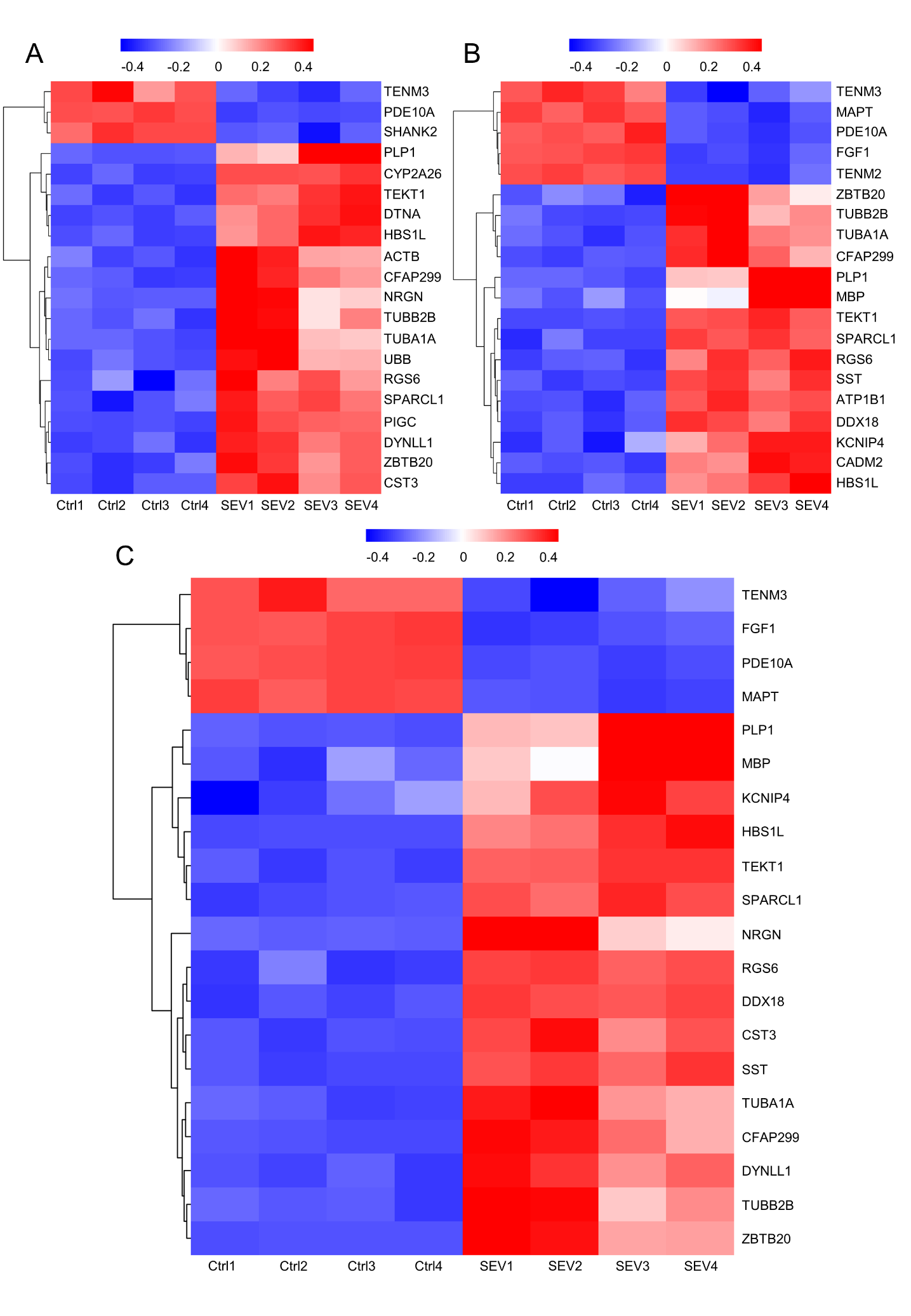
**

(A, B) Heatmap of Different genes expressed in the CNR1+neighborhood (A) and LHX6+ neighborhood (B) in the hippocampus of macaque after sevoflurane exposures. (C) Differentially expressed genes in the SST+ cluster in the inhibitory neuron of the macaque hippocampus after sevoflurane exposures, visualized in heatmap.

**Figure S2.
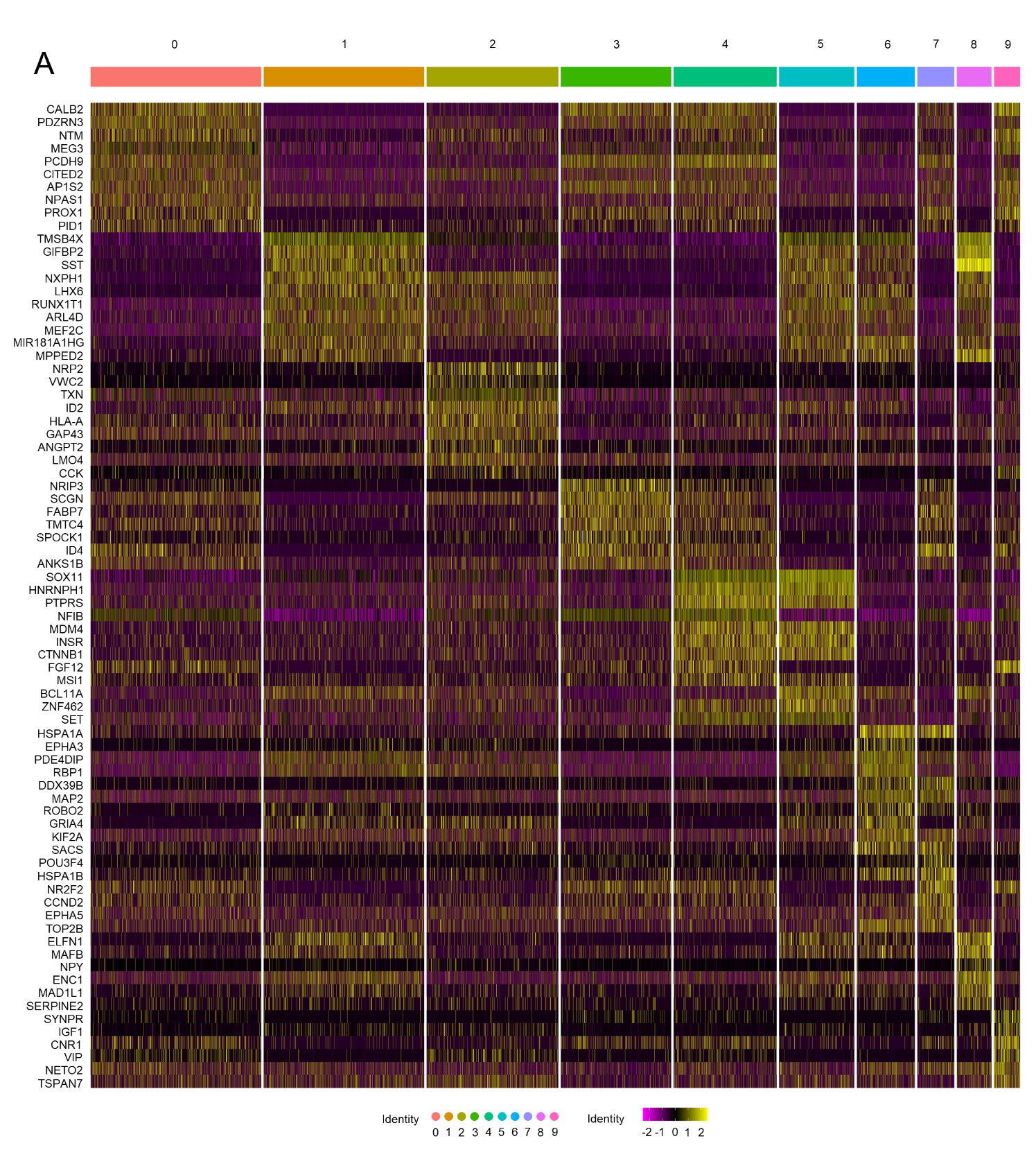
**(A) Heatmap of different genes expressed in the ten subcluster of the inhibitory neuron in the hippocampus of the developing human fetus.
